# Supplementary material for: How to Improve the Reactive Strength Index among Male Athletes? A Systematic Review with Meta-Analysis
Source: Healthcare (Basel). 2022 Mar 22;10(4):593. doi: 10.3390/healthcare10040593 (PMC9031107; doi:10.3390/healthcare10040593)
Supplement: Supplementary file 1 [file healthcare-10-00593-s001.zip › healthcare-1640679-supplementary.pdf]

healthcare

MDPI

SUPPLEMENTARY FILE

CONTENTS

Supplemental Material S1 Comprehensive search strategy

Supplemental Material S2 Risk of bias assessment using the Revised Cochrane risk-of-bias tool for randomized trials (RoB 2.0) scale

Supplemental Material S3 Risk of bias assessment using the Risk of Bias in Non-randomized Studies - of Interventions (ROBINS-I) tool

Supplemental Material S4 Characteristics of the included studies, interventions, and outcomes

Supplemental Material S5 Meta-analyses of all study designs

Supplemental Material S1. Comprehensive search strategy

ALL (("reactive strength index" OR "reactive-strength index" OR "reactive strength-index" OR "reactive-strength-index" OR "reactive strength" OR "reactive-strength" OR "reactive index" OR "reactive-index" OR “strength index” OR “strength-index” OR "RSI"))

AND

("player" OR "players" OR "athlete" OR "athletes" OR "sport" OR "sports" OR “sport participant” OR “sports participant” OR “sports participants” OR “sport participants” OR “modality” OR “modalities”)

Supplemental Material S2. Risk of bias assessment using the Revised Cochrane risk-of-bias tool for randomized trials (RoB 2.0) scale. Green means low risk of bias. Orange means some concerns. Red means high risk of bias.

Author (year)	Randomisation Process	Deviations From the Intended Interventions	Missing Outcomes	Measurement of The Outcome	Selection of Reported Results	Overall Risk of Bias
Hammami *et al* 2022 [71]	Low risk	Low risk	Low risk	Low risk	Low risk	Low risk
Murton *et al* 2021 [65]	Low risk	Low risk	Low risk	Low risk	Low risk	Low risk
Guo *et al* 2021 [66]	Low risk	Low risk	Low risk	Low risk	Low risk	Low risk
Ramirez-Campillo *et al* 2020 [61]	Low risk	Low risk	Low risk	Low risk	Low risk	Low risk
Fiorilli *et al* 2020 [44]	Low risk	Low risk	Low risk	Low risk	High risk	High risk
Bouguezzi *et al* 2020 [47]	Low risk	Low risk	High risk	Low risk	Low risk	High risk
Bouguezzi *et al* 2020 [67]	Low risk	Low risk	Low risk	Low risk	Low risk	Low risk
Orange *et al* 2019 [58]	Low risk	Low risk	Low risk	Some concerns	Low risk	Some concerns
Jeffreys *et al* 2019 [60]	Low risk	Low risk	Low risk	Low risk	Low risk	Low risk
Ramirez-Campillo *et al* 2019 [45]	Low risk	Some concerns	Low risk	Low risk	High risk	High risk
Ramirez-Campillo *et al* 2019 [46]	Some concerns	Low risk	Low risk	Low risk	High risk	High risk
Sporri *et al* 2018 [41]	Low risk	Low risk	Low risk	Low risk	Low risk	Low risk
Ramirez-Campillo *et al* 2018 [38]	Low risk	Low risk	Low risk	Low risk	Low risk	Low risk

Ramirez-Campillo *et al* 2018 [62]	**Low risk**	**Low risk**	**Low risk**	**Low risk**	**Low risk**	**Low risk**
Makhlouf *et al* 2018 [68]	**Low risk**	**Low risk**	**Low risk**	**Low risk**	**Some concerns**	**Some concerns**
Douglas *et al* 2018 [43]	**High risk**	**Low risk**	**Low risk**	**High risk**	**High risk**	**High risk**
Ciacci and Bartolomei 2018 [72]	**Low risk**	**Some concerns**	**Low risk**	**Low risk**	**Low risk**	**Some concerns**
Negra *et al* 2017 [70]	**Low risk**	**Low risk**	**Low risk**	**Low risk**	**Some concerns**	**Some concerns**
Dello Iacono *et al* 2017 [37]	**Low risk**	**Low risk**	**Low risk**	**Low risk**	**Low risk**	**Low risk**
Chaouachi *et al* 2017 [69]	**Low risk**	**Low risk**	**Low risk**	**Low risk**	**Low risk**	**Low risk**
Rosas *et al* 2016 [34]	**Low risk**	**Low risk**	**Low risk**	**Low risk**	**Low risk**	**Low risk**
Hammami *et al* 2016 [48]	**Low risk**	**Low risk**	**Low risk**	**Low risk**	**High risk**	**High risk**
Ramirez-Campillo *et al* 2015 [29]	**Low risk**	**Low risk**	**Low risk**	**Low risk**	**Low risk**	**Low risk**
Ramirez-Campillo *et al* 2015 [30]	**Some concerns**	**Some concerns**	**Low risk**	**Low risk**	**Low risk**	**Some concerns**
Ramirez-Campillo *et al* 2015 [31]	**Some concerns**	**Some concerns**	**Low risk**	**Low risk**	**Low risk**	**Some concerns**
Ramirez-Campillo *et al* 2015 [32]	**Low risk**	**Low risk**	**Low risk**	**Low risk**	**Low risk**	**Low risk**
Ramirez-Campillo *et al* 2014 [33]	**Some concerns**	**Some concerns**	**Low risk**	**Low risk**	**Low risk**	**Some concerns**
Lockie *et al* 2012 [49]	**High risk**	**High risk**	**Low risk**	**Low risk**	**Low risk**	**High risk**
Argus *et al* 2012 [35]	**High risk**	**Low risk**	**Low risk**	**Low risk**	**Some concerns**	**High risk**
Byrne *et al* 2010 [50]	**High risk**	**High risk**	**Low risk**	**Low risk**	**Low risk**	**High risk**
Salonikidis and Zafeiridis 2008 [51]	**High risk**	**Some concerns**	**Low risk**	**Low risk**	**Low risk**	**High risk**
Spinks *et al* 2007 [52]	**High risk**	**Some concerns**	**Low risk**	**Low risk**	**Low risk**	**High risk**
Young *et al* 1999 [53]	**Some concerns**	**Some concerns**	**High risk**	**Some concerns**	**Low risk**	**High risk**

Supplemental Material S3. Risk of bias assessment using the Risk of Bias in Non-randomized Studies - of Interventions (ROBINS-I) tool

**Author (year)**	**Confounding**	**Selection Bias**	**Classification Of Interventions**	**Reporting Bias**	**Deviations from Interventions**	**Missing Data**	**Measuring Outcomes**	**Overall Risk of Bias**
Dobbs *et al* 2020 [55]	Critical	Moderate	Low	Low	Low	Low	Low	Serious
Chaabene *et al* 2019 [57]	Serious	Serious	Moderate	Low	Low	Serious	Serious	Serious
Li *et al* 2019 [56]	Serious	Serious	Serious	Low	Low	Low	Low	Serious
Wee *et al* 2019 [42]	Critical	Critical	Low	Serious	Low	Low	Critical	Serious
Keiner *et al* 2018 [36]	Critical	Serious	Moderate	Low	Serious	Moderate	Moderate	Serious
Beattie *et al* 2017 [54]	Serious	Critical	Low	Low	Critical	Critical	Low	Critical

Supplemental Material S4. Characteristics of the included studies, interventions, and outcomes

Table S1. Randomized studies.

Authvor, year	N	Age	Sport	Intervention	Control	Measurement Equipment	Methodology	Results
Hammami *et al* 2022 [71]	27<br>G1: 14<br>C: 13	G1: 14.86 ± 0.52<br>C: 14.74 ± 0.36	Volleyball	Duration: 8 weeks<br>Type: Resistance elastic band training<br>Frequency: 2x week	Maintained training routines	Ergojump (Globus, Codogne, Italy)	Contact mat (Vertical Hops: jump height/contact time; hands on hips)	G1: 1.35 ± 0.15 ; 1.57 ± 0.16<br>C: 1.37 ± 0.26 ; 1.39 ± 0.42
Murton *et al* 2021 [65]	16<br>G1: 8<br>G2: 8	ALL: 18.0 ± 1.0	Rugby	Intervention 1<br>Duration: 4 weeks<br>Type: Flywheel Eccentric Overload Training Frequency: 3x/week<br>Intervention 2<br>Duration: 4 weeks<br>Type: Resistance Training<br>Frequency: 3x/week	N/A	ForceDecks (Vald Performance Pty Ltd., Brisbane, Australia)	Force Plate (DJ40: jump height / contact time; hands on hips)	G1: 1.94 ± 0.40 ; 2.07 ± 0.42<br>G2: 1.97 ± 0.35 ; 2.22 ± 0.47
Guo *et al* 2021 [66]	16<br>G1: 8<br>G2: 8	G1: 20.5 ± 1.1<br>G2: 19.1 ± 2.2	Badminton	Intervention 1<br>Duration: 6 weeks<br>Type: Balance plyometric training<br>Frequency: 3x/week<br>Intervention 2<br>Duration: 6 weeks<br>Type: Plyometric training<br>Frequency: 3x/week	N/A	Kistler (Quattro Jump, Winterhur, Switzerland)	Force Plate (DJ30: jump height / contact time; hands on hips)	G1: 1.27 ± 0.14 ; 1.50 ± 0.12<br>G2: 1.22 ± 0.12 ; 1.35 ± 0.15
Ramirez-Campillo *et al* 2020 [61]	38<br>G1: 12<br>G2: 14<br>C: 12	G1: 16.9 ± 0.7<br>G2: 17.1 ± 0.3<br>C: 17.1 ± 0.5	Soccer	Intervention 1<br>Duration: 7 weeks<br>Type: Plyometric training before regular soccer practice<br>Frequency: 2x/week<br>Intervention 2<br>Duration: 7 weeks<br>Type: Plyometric training after regular soccer practice<br>Frequency: 2x/week	Maintained training routines	Ergojump (Globus, Codogne, Italy)	Contact mat (DJ20: jump height / contact time; hands on hips)	G1: 1.77 ± 0.20 ; 2.11 ± 0.22<br>G2: 1.76 ± 0.32 ; 1.85 ± 0.31<br>C: 1.74 ± 0.28 ; 1.76 ± 0.31

Fiorilli *et al* 2020 [44]	34<br>G1: 16<br>G2: 18	G1: 13.36 ± 0.80<br>G2: 13.21 ± 1.21	Soccer	Intervention 1<br>Duration: 6 weeks<br>Type: Plyometric training<br>Frequency: 2x/week<br>Intervention 2<br>Duration: 6 weeks<br>Type: Flywheel Eccentric Overload Training<br>Frequency: 2x/week	N/A	Optojump (Microgate, Bolzano, Italy)	Photoelectric system (7 Hop Test: average jump height / average ground contact time; free arms)	G1: 0.70 ± 0.30 ; 0.76 ± 0.19<br>G2: 0.84 ± 0.18 ; 0.83 ± 0.18
Bouguezzi *et al* 2020 [47]	30<br>G1: 15<br>G2: 15	G1: 11.32 ± 0.27<br>G2: 12.27 ± 0.33	Soccer	Intervention 1<br>Duration: 8 weeks<br>Type: Plyometric training<br>Frequency: 1x/week<br>Intervention 2<br>Duration: 8 weeks<br>Type: Plyometric training<br>Frequency: 2x/week	N/A	Optojump (Microgate, Bolzano, Italy)	Photoelectric system (5 Hop Test: average jump height / average ground contact time; free arms)	G1: 0.72 ± 0.29 ; 1.15 ± 0.33<br>G2: 0.79 ± 0.21 ; 1.10 ± 0.27
Bouguezzi *et al* 2020 [67]	26<br>G1: 13<br>G2: 13	G1: 11.2 ± 0.5<br>G2: 11.3 ± 0.6	Soccer	Intervention 1<br>Duration: 8 weeks<br>Type: SSC-based using countermovement jump<br>Frequency: 2x week<br>Intervention 2<br>Duration: 8 weeks<br>Type: Non-SSC-based using squat jump<br>Frequency: 2x week	N/A	Optojump (Microgate, Bolzano, Italy)	Photoelectric system (Vertical Hops: jump height / contact time; hands on hips)	G1: 0.69 ± 0.22 ; 0.97 ± 0.26<br>G2: 0.68 ± 0.25 ; 0.67 ± 0.26
Orange *et al* 2019 [58]	27<br>G1: 12<br>G2: 15	ALL: 17.0 ± 1.0	Rugby	Intervention 1<br>Duration: 7 weeks<br>Type: Velocity-based training<br>Frequency: 2x week<br>Intervention 2<br>Duration: 7 weeks<br>Type: Percentage-based training<br>Frequency: 2x week	N/A	Optojump (Microgate, Bolzano, Italy)	Photoelectric system (DJ30: jump height / contact time; hands on hips)	G1: 1.34 ± 0.36 ; 1.35 ± 0.31<br>G2: 1.16 ± 0.18 ; 1.28 ± 0.20
Ramirez-Campillo *et al* 2019 [45]	39<br>G1: 19<br>C: 20	G1: 13.2 ± 1.8<br>C: 13.5 ± 1.9	Soccer	Duration: 7 weeks<br>Type: DJ training<br>Frequency: 2x week	Maintained training routines	Ergojump (Globus, Codogne, Italy)	Contact mat (DJ20: jump height /	G1: 1.07 ± 0.43 ; 1.24 ± 0.38<br>C: 1.09 ± 0.41 ; 1.03 ± 0.38

							contact time; hands on hips)	
Ramirez-Campillo *et al* 2019 [46]	26<br>G1: 7<br>G2: 7<br>G3: 6<br>G4: 6	G1: 10.9 ± 0.8<br>G2: 11.2 ± 1.4<br>G3: 15.0 ± 0.9<br>G4: 15.6 ± 1.3	Soccer	Intervention 1<br>Duration: 24 weeks<br>Type: Plyometric training with 30 s inter-set intervals (pre-PHV)<br>Frequency: 2x week<br>Intervention 2<br>Duration: 24 weeks<br>Type: Plyometric training with 120 s inter-set intervals (pre-PHV)<br>Frequency: 2x week<br>Intervention 3<br>Duration: 24 weeks<br>Type: Plyometric training with 30 s inter-set intervals (post-PHV)<br>Frequency: 2x week<br>Intervention 4<br>Duration: 24 weeks<br>Type: Plyometric training with 120 s inter-set intervals (post-PHV)<br>Frequency: 2x week	N/A	Ergojump (Globus, Codogne, Italy)	Contact mat (DJ20: jump height / contact time; hands on hips)	G1: 0.87 ± 0.5 ; 1.13 ± 0.4<br>G2: 0.98 ± 0.2 ; 1.32 ± 0.2<br>G3: 1.28 ± 0.7 ; 1.80 ± 0.7<br>G4: 1.22 ± 0.5 ; 1.65 ± 0.4
Jeffreys *et al* 2019 [60]	29<br>G1: 10<br>G2: 9<br>C: 10	ALL: 20.3 ± 1.6	Rugby	Intervention 1<br>Duration: 6 weeks<br>Type: Low Volume Plyometric Training<br>Frequency: 2x/week<br>Intervention 2<br>Duration: 6 weeks<br>Type: High Volume Plyometric Training<br>Frequency: 2x/week	Maintained training routines	Kistler (Quattro Jump, Winterhur, Switzerland)	Force Plate (DJ45: jump height / contact time; hands on hips)	G1: 1.33 ± 0.40 ; 1.45 ± 0.38<br>G2: 1.26 ± 0.36 ; 1.36 ± 0.38<br>C: 0.91 ± 0.24 ; 0.85 ± 0.22
Sporri *et al* 2018 [41]	21<br>G1: 11<br>C: 9	ALL: 22.2 ± 2.5	Team Sports	Duration: 8 weeks<br>Type: Aqua-based plyometric	Maintained training routines	Onspot (Wollongong, Australia)	Force plate (DJ50: jump height /	G1: 9.90 ± 4.00 ; 11.4 ± 4.30<br>C: 13.3 ± 4.00 ; 12.2 ± 4.50

				training<br>Frequency: 3x week			contact time; hands on hips)	
Ciacci and Bartolomei 2018 [72]	58<br>G1: 10<br>G2: 10<br>G3: 9<br>G4: 12<br>G5: 8<br>G6: 9	G1: 24.3 ± 7.0<br>G2: 17.5 ± 0.5<br>G3: 15.7 ± 0.5<br>G4: 20.1 ± 1.6<br>G5: 17.4 ± 0.5<br>G6: 14.8 ± 0.4	Basketball	Interventions 1, 2 and 3<br>Duration: 12 weeks<br>Type: Hang Clean Training<br>Frequency: 2x week<br>Interventions 4, 5 and 6<br>Duration: 12 weeks Type: Half Squat Training<br>Frequency: 2x week	N/A	Ergotester PRO (Globus, Treviso, Italy)	Contact mat (CMJ: contact time / time to takeoff; hands on hips)	G1: 2.58 ± 0.44 ; 2.67 ± 0.56<br>G2: 2.40 ± 0.37 ; 2.60 ± 0.47<br>G3: 2.62 ± 0.43 ; 2.86 ± 0.36<br>G4: 2.70 ± 0.30 ; 2.72 ± 0.36<br>G5: 2.52 ± 0.32 ; 2.69 ± 0.46<br>G6: 2.01 ± 0.39 ; 2.21 ± 0.25
Ramirez-Campillo *et al* 2018 [38]	73<br>G1: 25<br>G2: 24<br>C: 24	G1: 13.9 ± 1.9<br>G2: 13.1 ± 1.7<br>C: 13.7 ± 1.6	Soccer	Intervention 1<br>Duration: 7 weeks<br>Type: Plyometric training from a fixed box height of 30 cm<br>Frequency: 2x/week<br>Intervention 2<br>Duration: 7 weeks<br>Type: Plyometric training from an individualized box height<br>Frequency: 2x/week	Maintained training routines	Ergojump (Globus, Codogne, Italy)	Contact mat (DJ20: flight time / contact time; hands on hips)	G1: 1.1 ± 0.5 ; 1.3 ± 0.5<br>G2: 1.1 ± 0.5 ; 1.5 ± 0.5<br>C: 1.1 ± 0.5 ; 1.2 ± 0.4
Douglas *et al* 2018 [43]	14<br>G1: 7<br>C: 7	ALL: 19.4 ± 0.8	Rugby	Duration: 12 weeks<br>Type: Accentuated eccentric resistance training<br>Frequency: 2x/week strength + 1x/week power	Duration: 12 weeks<br>Type: Traditional resistance training<br>Frequency: 2x/week strength + 1x/week power	AMTI (Watertown, MA, USA)	Force plate (DJ50: flight time / contact time; hands on hips)	G1: 2.29 ± 0.32 ; 2.41 ± 0.45<br>C: 1.94 ± 0.31 ; 1.97 ± 0.56
Ramirez-Campillo *et al* 2018 [62]	76<br>G1: 38<br>C: 38	10-16	Soccer	Duration: 7 weeks<br>Type: Plyometric training<br>Frequency: 2x/week	Maintained training routines	Ergojump (Globus, Codogne, Italy)	Contact mat (DJ20: jump height / contact time; hands on hips)	G1: 23.3% ± 17.3%<br>C: -1.7% ± 13.2%
Makhlouf *et al* 2018 [68]	57<br>G1: 21<br>G2: 20<br>C: 16	G1: 11.1 ± 0.8<br>G2: 11.3 ± 0.9<br>C: 11.0 ± 0.8	Soccer	Intervention 1<br>Duration: 8 weeks<br>Type: Balance-Plyometric Training<br>Frequency: 2x/week	Maintained training routines	Ergojump (Globus, Codogne, Italy)	Contact mat (Vertical Hops: flight time / contact	G1: 0.8 ± 0.3 ; 1.1 ± 0.2<br>G2: 0.8 ± 0.2 ; 1.1 ± 0.3<br>C: 0.8 ± 0.3 ; 0.9 ± 0.3

				Intervention 2<br>Duration: 8 weeks<br>Type: Agility-Plyometric Training<br>Frequency: 2x/week			time; hands on hips)	
Chaouachi *et al* 2017 [69]	26<br>G1: 13<br>G2: 13	G1: 13.9 ± 0.27<br>G2: 13.8 ± 0.32	Soccer	Intervention 1<br>Duration: 8 weeks<br>Type: Alternated a series of paired individual balance and plyometric exercises<br>Frequency: 2x week<br>Intervention 2<br>Duration: 8 weeks<br>Type: A block of balance exercises prior to a block of plyometric exercises<br>Frequency: 2x week	N/A	Ergojump (Globus, Codogne, Italy)	Contact mat (Triple Hop Test: jump height / contact time; hands on hips)	G1: 0.8 ± 0.3 ; 1.21 ± 0.19<br>G2: 0.79 ± 0.25 ; 1.31 ± 0.27
Dello Iacono *et al* 2017 [37]	18<br>G1: 9<br>G2: 9	ALL: 23.4 ± 4.6	Team handball	Intervention 1<br>Duration: 10 weeks<br>Type: Vertical-oriented drop jump training<br>Frequency: 2x week<br>Intervention 2<br>Duration: 10 weeks<br>Type: Horizontal-oriented drop jump training<br>Frequency: 2x week	N/A	Kistler (Quattro Jump, Winterhur, Switzerland)	Force Plate (CMJ: jump height / contact time; hands on hips)	G1: 0.60 ± 0.05 ; 7.2 %<br>G2: 0.59 ± 0.06 ; 2.1 %
Negra *et al* 2017 [70]	33<br>G1: 17<br>G2: 16	G1: 12.1 ± 0.5<br>G2: 12.2 ± 0.6	Soccer	Intervention 1<br>Duration: 8 weeks<br>Type: Plyometric training on stable surfaces<br>Frequency: 2x/week<br>Intervention 2<br>Duration: 8 weeks<br>Type: Plyometric training on stable and unstable surfaces<br>Frequency: 2x/week	N/A	Optojump (Microgate, Bolzano, Italy)	Photoelectric system (Vertical Hops: jump height / contact time; hands on hips)	G1: 1.1 ± 0.3 ; 1.3 ± 0.1<br>G2: 1.5 ± 0.3 ; 1.1 ± 0.1
Hammami *et al* 2016 [48]	24<br>G1: 12<br>G2: 12	G1: 12.7 ± 0.3<br>G2: 12.5 ± 0.3	Soccer	Intervention 1<br>Duration: 8 weeks<br>Type: 4 weeks of balance	N/A	Ergojump (Globus, Codogne, Italy)	Force Plate (Vertical Hops: jump	G1: 1.1 ± 0.1 ; 1.4 ± 0.3<br>G2: 0.89 ± 0.15 ; 0.94 ± 0.07

				training first followed by 4 weeks of plyometric training<br>Frequency: 2x week<br>Intervention 2<br>Duration: 8 weeks<br>Type: 4 weeks of plyometric training first followed by 4 weeks of balance training<br>Frequency: 2x week			height / contact time; hands on hips)	
Rosas *et al* 2016 [34]	63<br>G1: 21<br>G2: 21<br>C: 21	G1: 12.3 ± 2.3<br>G2: 12.1 ± 2.1<br>C: 12.0 ± 2.2	Soccer	Intervention 1<br>Duration: 6 weeks<br>Type: Plyometric training<br>Frequency: 2x week<br>Intervention 2<br>Duration: 6 weeks<br>Type: Hand-held loaded plyometric training<br>Frequency: 2x week	Maintained training routines	Ergojump (Globus, Codogne, Italy)	Contact mat (DJ20: jump height / contact time; hands on hips)	G1: 1.05 ± 0.4 ; 1.14 ± 0.4*<br>G2: 0.95 ± 0.5 ; 1.10 ± 0.5*<br>C: 1.19 ± 0.4 ; 1.21 ± 0.4*
Ramirez-Campillo *et al* 2015 [29]	166<br>G1: 54<br>G2: 57<br>C: 55	G1: 14.2 ± 2.2<br>G2: 14.1 ± 2.2<br>C: 14.0 ± 2.3	Soccer	Intervention 1<br>Duration: 6 weeks<br>Type: Plyometric training with 24h of rest between sessions<br>Frequency: 2x week<br>Intervention 2<br>Duration: 6 weeks<br>Type: Plyometric training with 48h of rest between sessions<br>Frequency: 2x week	Maintained training routines	Ergojump (Globus, Codogne, Italy)	Contact mat (DJ20: jump height / contact time; hands on hips)	G1: 1.32 ± 0.40 ; 1.46 ± 0.40*<br>G2: 1.37 ± 0.39 ; 1.51 ± 0.37*<br>C: 1.31 ± 0.42 ; 1.32 ± 0.41*
Ramirez-Campillo *et al* 2015 [30]	40<br>G1: 10<br>G2: 10<br>G3: 10<br>C: 10	G1: 11.6 ± 1.4<br>G2: 11.4 ± 1.9<br>G3: 11.2 ± 2.3<br>C: 11.4 ± 2.4	Soccer	Intervention 1<br>Duration: 6 weeks<br>Type: Vertical plyometric<br>Frequency: 2x week<br>Intervention 2<br>Duration: 6 weeks<br>Type: Horizontal plyometric<br>Frequency: 2x week<br>Intervention 3<br>Duration: 6 weeks<br>Type: Combined plyometric<br>Frequency: 2x week	Maintained training routines	Ergojump (Globus, Codogne, Italy)	Contact mat (DJ20: jump height / contact time; hands on hips)	G1: 1.26 ± 0.20 ; 1.45 ± 0.20*<br>G2: 1.32 ± 0.36 ; 1.47 ± 0.32*<br>G3: 1.17 ± 0.41 ; 1.38 ± 0.49*<br>C: 1.42 ± 0.41 ; 1.46 ± 0.39*

Ramirez-Campillo *et al* 2015 [31]	54<br>G1: 12<br>G2: 16<br>G3: 12<br>C: 14	G1: 11.0 ± 2.0<br>G2: 11.6 ± 1.7<br>G3: 11.6 ± 2.7<br>C: 11.2 ± 2.4	Soccer	Intervention 1<br>Duration: 6 weeks<br>Type: Bilateral plyometric training<br>Frequency: 2x week<br>Intervention 2<br>Duration: 6 weeks<br>Type: Unilateral plyometric training<br>Frequency: 2x week<br>Intervention 3<br>Duration: 6 weeks<br>Type: Bilateral + unilateral plyometric training<br>Frequency: 2x week	Maintained training routines	Ergojump (Globus, Codogne, Italy)	Contact mat (DJ20: jump height / contact time; hands on hips)	G1: 1.3 ± 0.5 ; 1.6 ± 0.4*<br>G2: 1.2 ± 0.3 ; 1.3 ± 0.3*<br>G3: 1.2 ± 0.5 ; 1.4 ± 0.5*<br>C: 1.4 ± 0.4 ; 1.4 ± 0.4*
Ramirez-Campillo *et al* 2015 [32]	24<br>G1: 8<br>G2: 8<br>C: 8	G1: 12.8 ± 2.8<br>G2: 13.0 ± 2.1<br>C: 13.0 ± 1.9	Soccer	Intervention 1<br>Duration: 6 weeks<br>Type: Plyometric with progressive increase in volume<br>Frequency: 2x week<br>Intervention 2<br>Duration: 6 weeks<br>Type: Plyometric without progressive increase in volume<br>Frequency: 2x week	Maintained training routines	Ergojump (Globus, Codogne, Italy)	Contact mat (DJ20: jump height / contact time; hands on hips)	G1: 0.072 ± 0.034 ; 0.092 ± 0.022*<br>G2: 0.062 ± 0.022 ; 0.076 ± 0.038*<br>C: 0.080 ± 0.029; 0.084 ± 0.048*
Ramirez-Campillo *et al* 2014 [33]	76<br>G1: 38<br>C: 38	G1: 13.2 ± 1.8<br>C: 13.2 ± 1.8	Soccer	Duration: 7 weeks<br>Type: Plyometric training<br>Frequency: 2x week	Maintained training routines	Ergojump (Globus, Codogne, Italy)	Contact mat (DJ20: jump height / contact time; hands on hips)	G1: 1.04 ± 0.40 ; 1.23 ± 0.35*<br>C: 1.01 ± 0.38 ; 0.98 ± 0.35*
Lockie *et al* 2012 [49]	35<br>G1: 9<br>G2: 8<br>G3: 9<br>G4: 9	ALL: 23.1 ± 4.2	Field sports	Intervention 1<br>Duration: 6 weeks<br>Type: Free sprint training<br>Frequency: 2x week<br>Intervention 2<br>Duration: 6 weeks<br>Type: Weight training<br>Frequency: 2x week<br>Intervention 3	N/A	Onspot (Wollongong, Australia)	Contact mat (DJ40: jump height / contact time; hands on hips)	G1: 1.07 ± 0.38 ; 1.27 ± 0.35<br>G2: 1.24 ± 0.21 ; 1.39 ± 0.31<br>G3: 1.31 ± 0.28 ; 1.44 ± 0.22<br>G4: 1.16 ± 0.21 ; 1.31 ± 0.26

				Duration: 6 weeks<br>Type: Plyometric training<br>Frequency: 2x week<br>Intervention 4<br>Duration: 6 weeks<br>Type: Resisted sprint training<br>Frequency: 2x week				
Argus *et al* 2012 [35]	18<br>G1: 9<br>G2: 9	G1: 23 ± 2<br>G2: 25 ± 2	Rugby	Intervention 1<br>Duration: 4 weeks<br>Type: High volume-load (strength-power) resistance training<br>Frequency: 2x week<br>Intervention 2<br>Duration: 4 weeks<br>Type: Low volume-load (speed-power) resistance training<br>Frequency: 2x week	N/A	400 Series Performance (Fitness Technology, Australia)	Force plate (DJ30: jump height / contact time; hands on hips)	G1: 1.83 ± 0.27 ; 0.8 %<br>G2: 1.86 ± 0.30 ; 3.4 %
Byrne *et al* 2010 [50]	19<br>G1: 6<br>G2: 6<br>C: 7	G1: 23.8 ± 7.6<br>G2: 20.8 ± 1.9<br>C: 18.7 ± 7.6	Basketball, rugby, hurling, Gaelic football and soccer	Intervention 1<br>Duration: 8 weeks<br>Type: Maximum jump height training method<br>Frequency: 2x week<br>Intervention 2<br>Duration: 8 weeks<br>Type: Reactive strength index training method<br>Frequency: 2x week	Maintained training routines	Power timer clock (Newtest, Oulu, Finland)	Force plate (3 Bounce DJ30: jump height / contact time; hands on hips)	G1: 1.35 ± 0.22 ; 1.67 ± 0.34<br>G2: 1.51 ± 0.49 ; 1.68 ± 0.58<br>C: 1.26 ± 0.14 ; 1.31 ± 0.13
Salonikidis and Zafeiridis 2008 [51]	64<br>G1: 16<br>G2: 16<br>G3: 16<br>C: 16	ALL: 21.1 ± 1.3	Tennis	Intervention 1<br>Duration: 9 weeks<br>Type: Plyometric training<br>Frequency: 3x week<br>Intervention 2<br>Duration: 9 weeks<br>Type: Tennis-drills training<br>Frequency: 3x week<br>Intervention 3	Maintained training routines	AMD Co. Ltd., LC 4204-K600 (1-D, Saitawa, Japan)	Load cell (DJ20: jump height / contact time; hands on hips)	G1: 125.3 ± 45.2 ; 150.0 ± 46.9<br>G2: 122.0 ± 25.0 ; 133.4 ± 27.1<br>G3: 128.8 ± 42.5 ; 153.7 ± 44.1<br>C: 130.8 ± 18.2 ; 124.2 ± 29.0

				Duration: 9 weeks<br>Type: Combined training<br>Frequency: 3x week				
Spinks *et al* 2007 [52]	30<br>G1: 10<br>G2: 10<br>C: 10	ALL: 21.8 ± 4.2	Soccer, rugby union and Australian football	Intervention 1<br>Duration: 8 weeks<br>Type: Sprint training + resisted sprint training<br>Frequency: 2x week<br>Intervention 2<br>Duration: 8 weeks<br>Type: Nonresisted sprint training<br>Frequency: 2x week	Maintained training routines	Onspot (Wollongong, Australia)	Force plate (DJ50: jump height / contact time; hands on hips)	G1: 117.2 ± 27.1 ; 137.3 ± 27.7<br>G2: 112.2 ± 18.9 ; 121.8 ± 21.3<br>C: 127.2 ± 36.2 ; 128.7 ± 25.1
Young *et al* 1999 [53]	25<br>G1: 11<br>G2: 5<br>C: 9	ALL: 19-34	Track and field, basketball and soccer	Intervention 1<br>Duration: 6 weeks<br>Type: DJ for height<br>Frequency: 3x week<br>Intervention 2<br>Duration: 6 weeks<br>Type: DJ for height and minimum contact time<br>Frequency: 3x week	Maintained training routines	?	Contact mat (DJ30: jump height / contact time; hands on hips)	G1: 180 ± 26 ; 182 ± 37<br>G2: 203 ± 50 ; 234 ± 32<br>C: 210 ± 45 ; 201 ± 65

C = control, CMJ = countermovement jump, DJ = drop jump, G = group, N = number, ? = unknown, * = obtained from authors.

Table S2. Non-randomized studies.

Author, year	N	Age	Sport	Intervention	Control	Measurement Equipment	Methodology	Results
Dobbs *et al* 2020 [55]	39<br>Pre-PHV G1: 14<br>Post-PHV G2: 7<br>Pre-PHV C1: 10<br>Post-PHV C2: 7	ALL: 9-17	?	Duration: 12 weeks<br>Type: Neuromuscular training<br>Frequency: 2x/week strength training	Maintained training routines	AMTI OR6-5 (Watertown, MA, USA)	Force plate (CMJ: jump height / time to takeoff; hands on hips)	G1: 0.23 ± 0.09 ; 0.27 ± 0.09<br>G2: 0.32 ± 0.08 ; 0.41 ± 0.07<br>C1: 0.24 ± 0.04 ; 0.21 ± 0.05<br>C2: 0.35 ± 0.15 ; 0.34 ± 0.12
Chaabene *et al* 2019 [57]	15	13.2 ± 1.3	Weightlifting	Duration: 5 months<br>Type: Weightlifting season<br>Frequency: ?	N/A	Optojump (Microgate, Bolzano, Italy)	Photoelectric system (DJ: jump height / contact time; hands on hips)	1.0 ± 0.2 ; 1.0 ± 0.2

Li *et al* 2019 [56]	28<br>G1: 10<br>G2: 19<br>C: 9	G1: 20.2 ± 1.0<br>G2: 21.2 ± 1.5<br>C: 20.8 ± 1.2	Distance running	Intervention 1<br>Duration: 8 weeks<br>Type: Complex training (afternoon) + endurance training (morning)<br>Frequency: 3x/week + 6x/week<br>Intervention 2<br>Duration: 8 weeks<br>Type: Heavy strength training (afternoon) + endurance training (morning)<br>Frequency: 3x/week + 6x/week	Duration: 8 weeks<br>Type: Strength endurance training (afternoon) + endurance training (morning)<br>Frequency: 3x/week + 6x/week	Kistler Quattro Jump, Winterhur, Switzerland	Force Plate (DJ50: jump height / contact time; hands on hips)	G1: 59.05 ± 11.63 ; 70.80 ± 15.69<br>G2: 61.57 ± 12.95 ; 66.71 ± 8.53<br>C: 62.11 ± 12.04 ; 62.91 ± 12.07
Wee *et al* 2019 [42]	18<br>G1: 9<br>C: 9	ALL: 20.0 ± 1.0	Badminton	Duration: 4 weeks<br>Type: Badminton specific training<br>Frequency: 2x/week	Maintained training routines	Bertec FP4060-10-4000	Force plate (DJ30: jump height / contact time; hands on hips)	G1: 1.16 ± 0.39 ; 1.32 ± 0.42<br>C: 1.06 ± 0.14 ; 1.11 ± 0.40
Keiner *et al* 2018 [36]	114<br>G1: 62<br>C: 52	ALL: 13-19	Soccer	Duration: 2 years<br>Type: Strength training<br>Frequency: 2x/week	Maintained training routines	Refitronic, Schmitten, Germany	Contact mat (DJ24: jump height / contact time; hands on hips)	?
Beattie *et al* 2017 [54]	20<br>G1: 11<br>C: 9	G1: 27.4 ± 7.2<br>C: 29.5 ± 10.0	Distance running	Duration: 40 weeks<br>Type: Endurance training and strength training<br>Frequency: 2x/week	Maintained training routines	AMTI OR6-5 (Watertown, MA, USA)	Force plate (DJ30: jump height / contact time; hands on hips)	G1: 1.10 ± 0.28 ; 1.26 ± 0.33<br>C: 1.28 ± 0.31 ; 1.16 ± 0.12

C = control, CMJ = countermovement jump, DJ = drop jump, G = group, N = number, ? = unknown.

Supplemental Material S5. Meta-analyses of all study designs.

a)

Study or Subgroup	Experimental Mean	Experimental SD	Experimental Total	Control Mean	Control SD	Control Total	Weight	Std. Mean Difference IV, Random, 95% CI
**Adults Plyometric (Only Randomized Studies)**								
Byrne et al 2010 (G1)	1.67	0.34	6	1.31	0.13	7	1.7%	1.35 [0.09, 2.60]
Byrne et al 2010 (G2)	1.68	0.58	6	1.31	0.13	7	2.0%	0.85 [-0.30, 2.01]
Jeffreys et al 2019 (G1)	1.45	0.38	10	0.85	0.22	10	2.2%	1.85 [0.77, 2.94]
Jeffreys et al 2019 (G2)	1.36	0.38	9	0.85	0.22	10	2.2%	1.59 [0.53, 2.66]
Salonikidis & Zefeiridis 2008 (G1)	150	46.9	16	124.2	29	16	3.9%	0.65 [-0.07, 1.36]
Salonikidis & Zefeiridis 2008 (G2)	153.7	44.1	16	124.2	29	16	3.9%	0.77 [0.05, 1.49]
Young et al 1999 (G1)	182	27	11	201	65	9	2.9%	-0.38 [-1.27, 0.51]
Young et al 1999 (G2)	234	32	5	201	65	9	2.1%	0.55 [-0.57, 1.67]
**Subtotal (95% CI)**			**79**			**84**	**20.8%**	**0.84 [0.37, 1.32]**
Heterogeneity: Tau² = 0.22; Chi² = 13.60, df = 7 (P = 0.06); I² = 49%								
Test for overall effect: Z = 3.47 (P = 0.0005)								
**Youths Plyometric (Only Randomized Studies)**								
Makhlouf et al 2018	1.1	0.2	20	0.9	0.3	16	4.1%	0.78 [0.10, 1.47]
Ramirez-Campillo et al 2014	1.23	0.35	38	0.98	0.35	38	6.0%	0.71 [0.24, 1.17]
Ramirez-Campillo et al 2015a (G1)	1.46	0.4	54	1.32	0.41	55	6.9%	0.34 [-0.04, 0.72]
Ramirez-Campillo et al 2015a (G2)	1.51	0.37	57	1.32	0.41	55	7.0%	0.48 [0.11, 0.86]
Ramirez-Campillo et al 2015b (G1)	1.45	0.2	10	1.46	0.39	10	3.0%	-0.03 [-0.91, 0.85]
Ramirez-Campillo et al 2015b (G2)	1.47	0.32	10	1.46	0.39	10	3.0%	0.03 [-0.85, 0.90]
Ramirez-Campillo et al 2015b (G3)	1.38	0.49	10	1.46	0.39	10	3.0%	-0.17 [-1.05, 0.71]
Ramirez-Campillo et al 2015c (G1)	1.6	0.4	12	1.4	0.4	14	3.5%	0.48 [-0.30, 1.27]
Ramirez-Campillo et al 2015c (G2)	1.3	0.3	16	1.4	0.4	14	3.9%	-0.28 [-1.00, 0.44]
Ramirez-Campillo et al 2015c (G3)	1.4	0.5	12	1.4	0.4	14	3.5%	0.00 [-0.77, 0.77]
Ramirez-Campillo et al 2015d (G1)	0.092	0.022	8	0.084	0.048	8	2.5%	0.20 [-0.78, 1.19]
Ramirez-Campillo et al 2015d (G2)	0.076	0.038	8	0.084	0.048	8	2.5%	-0.17 [-1.16, 0.81]
Ramirez-Campillo et al 2018 (G1)	1.3	0.5	24	1.2	0.4	25	5.1%	0.22 [-0.34, 0.78]
Ramirez-Campillo et al 2018 (G2)	1.5	0.5	24	1.2	0.4	25	5.0%	0.65 [0.08, 1.23]
Ramirez-Campillo et al 2019	1.24	0.38	19	1.03	0.38	20	4.4%	0.54 [-0.10, 1.18]
Ramirez-Campillo et al 2020 (G1)	2.11	0.22	12	1.76	0.31	12	2.9%	1.26 [0.37, 2.15]
Ramirez-Campillo et al 2020 (G2)	1.85	0.31	14	1.76	0.31	12	3.5%	0.28 [-0.49, 1.06]
Rosas et al 2016 (G1)	1.14	0.4	21	1.21	0.4	21	4.7%	-0.17 [-0.78, 0.43]
Rosas et al 2016 (G2)	1.1	0.5	21	1.21	0.4	21	4.7%	-0.24 [-0.85, 0.37]
**Subtotal (95% CI)**			**390**			**388**	**79.2%**	**0.30 [0.13, 0.47]**
Heterogeneity: Tau² = 0.03; Chi² = 23.86, df = 18 (P = 0.16); I² = 25%								
Test for overall effect: Z = 3.45 (P = 0.0006)								
**Total (95% CI)**			**469**			**472**	**100.0%**	**0.40 [0.22, 0.58]**
Heterogeneity: Tau² = 0.09; Chi² = 43.97, df = 26 (P = 0.02); I² = 41%								
Test for overall effect: Z = 4.33 (P < 0.0001)								
Test for subgroup differences: Chi² = 4.41, df = 1 (P = 0.04), I² = 77.3%								

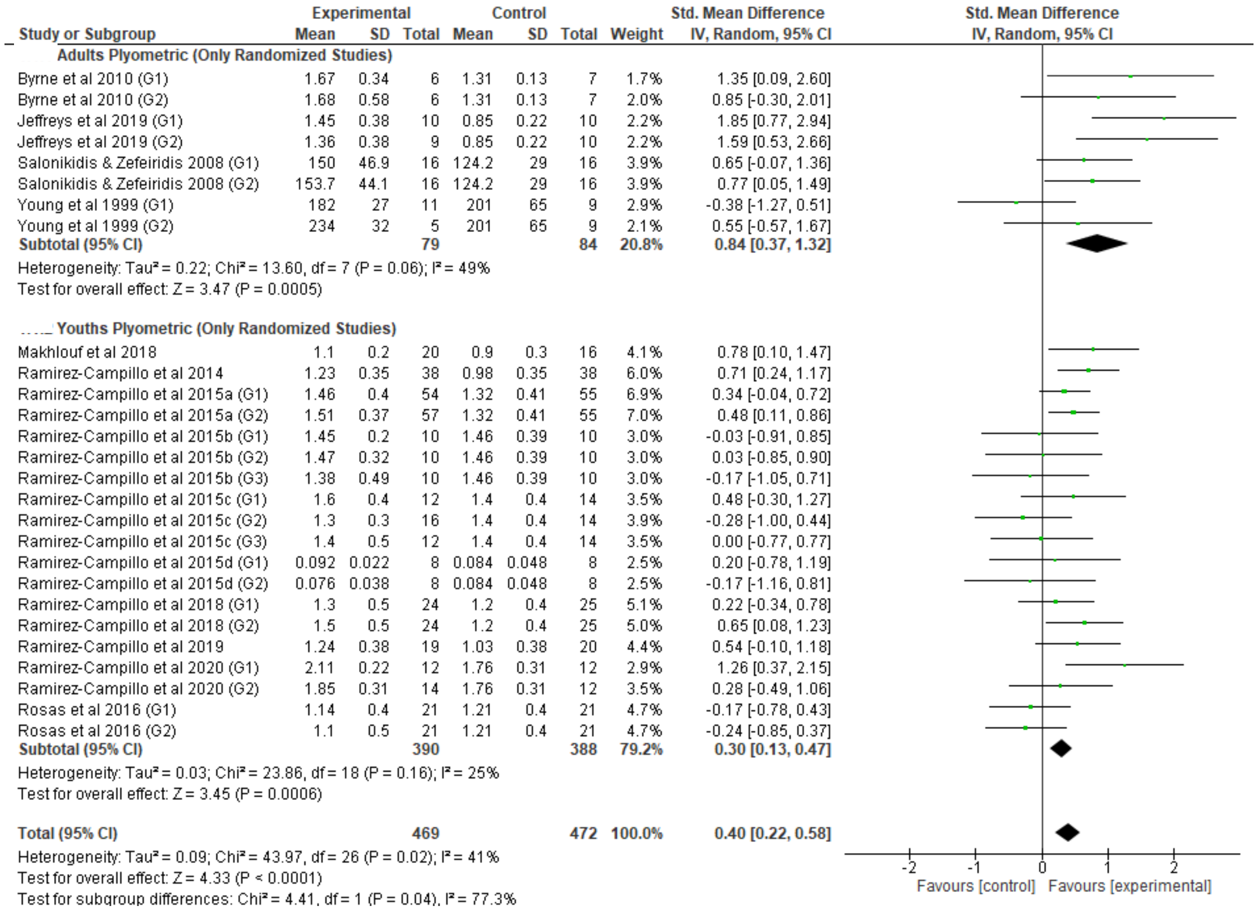

b)

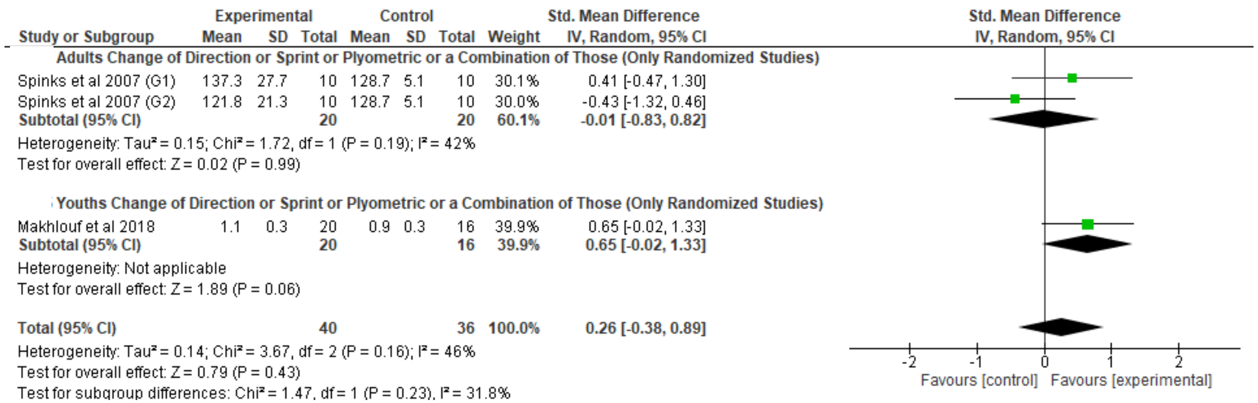

c)

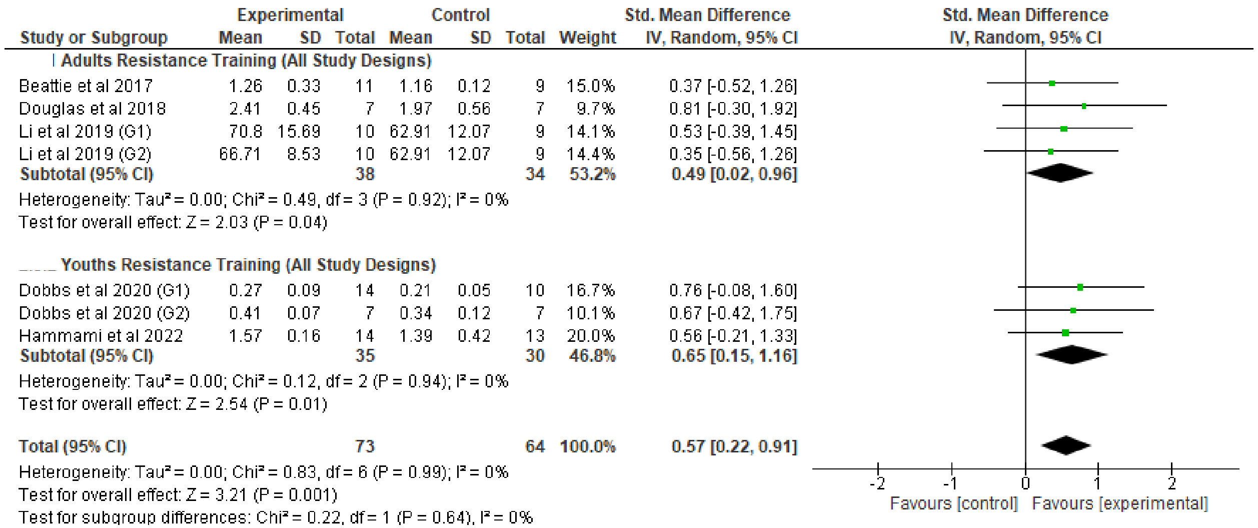

Figure S1. Forest plot of the overall standardized mean difference [95% CI] for each study (randomized and non-randomized) with control group (the size of the green dot corresponds to the weight of the study within the meta-analysis)with (**a**) a plyometric training intervention, (**b**) a combination of change of direction, sprint, or plyometric training intervention and (**c**) a resistance training intervention included in the meta-analysis.

a)

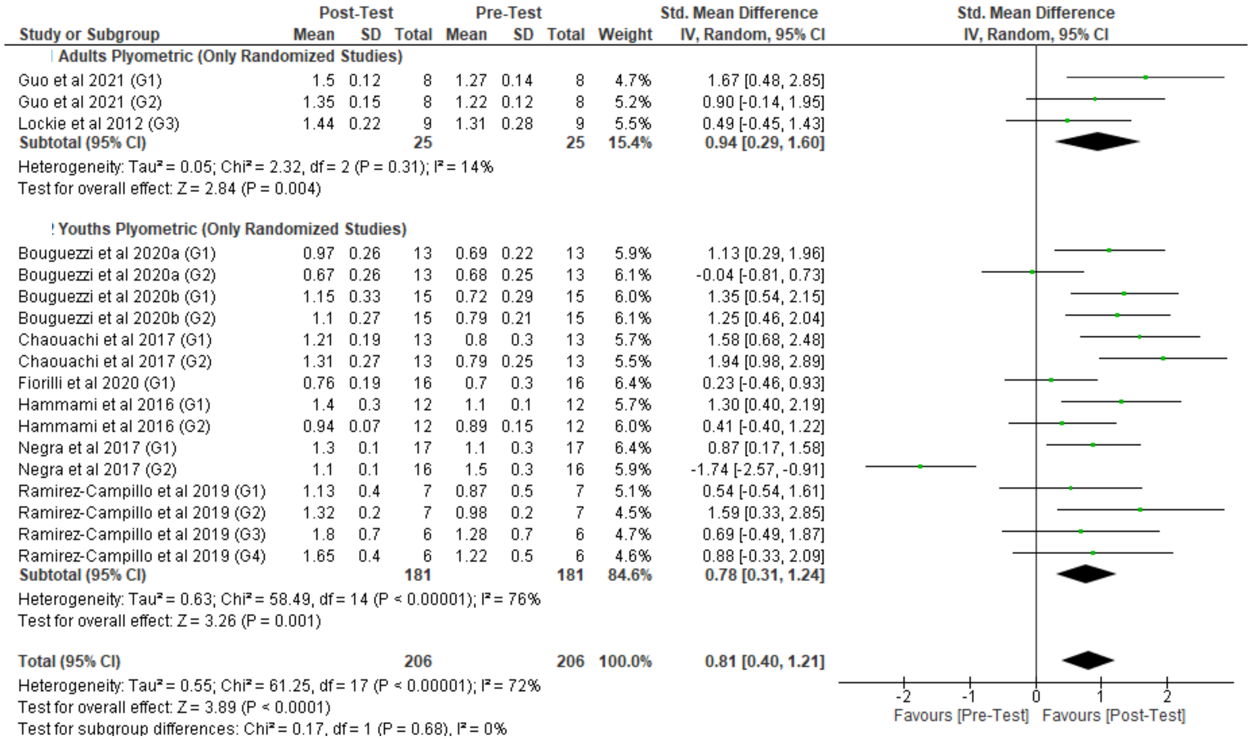
Post-Test
Pre-Test
Std. Mean Difference
Std. Mean Difference
Study or Subgroup Mean SD Total Mean SD Total Weight IV, Random, 95% CI IV, Random, 95% CI
Adults Plyometric (Only Randomized Studies)
Guo et al 2021 (G1) 1.5 0.12 8 1.27 0.14 8 4.7% 1.67 [0.48, 2.85]
Guo et al 2021 (G2) 1.35 0.15 8 1.22 0.12 8 5.2% 0.90 [-0.14, 1.95]
Lockie et al 2012 (G3) 1.44 0.22 9 1.31 0.28 9 5.5% 0.49 [-0.45, 1.43]
Subtotal (95% CI) 25 25 15.4% 0.94 [0.29, 1.60]
Heterogeneity: Tau² = 0.05; Chi² = 2.32, df = 2 (P = 0.31); I² = 14%
Test for overall effect: Z = 2.84 (P = 0.004)
Youths Plyometric (Only Randomized Studies)
Bouguezzi et al 2020a (G1) 0.97 0.26 13 0.69 0.22 13 5.9% 1.13 [0.29, 1.96]
Bouguezzi et al 2020a (G2) 0.67 0.26 13 0.68 0.25 13 6.1% -0.04 [-0.81, 0.73]
Bouguezzi et al 2020b (G1) 1.15 0.33 15 0.72 0.29 15 6.0% 1.35 [0.54, 2.15]
Bouguezzi et al 2020b (G2) 1.1 0.27 15 0.79 0.21 15 6.1% 1.25 [0.46, 2.04]
Chaouachi et al 2017 (G1) 1.21 0.19 13 0.8 0.3 13 5.7% 1.58 [0.68, 2.48]
Chaouachi et al 2017 (G2) 1.31 0.27 13 0.79 0.25 13 5.5% 1.94 [0.98, 2.89]
Fiorilli et al 2020 (G1) 0.76 0.19 16 0.7 0.3 16 6.4% 0.23 [-0.46, 0.93]
Hammami et al 2016 (G1) 1.4 0.3 12 1.1 0.1 12 5.7% 1.30 [0.40, 2.19]
Hammami et al 2016 (G2) 0.94 0.07 12 0.89 0.15 12 6.0% 0.41 [-0.40, 1.22]
Negra et al 2017 (G1) 1.3 0.1 17 1.1 0.3 17 6.4% 0.87 [0.17, 1.58]
Negra et al 2017 (G2) 1.1 0.1 16 1.5 0.3 16 5.9% -1.74 [-2.57, -0.91]
Ramirez-Campillo et al 2019 (G1) 1.13 0.4 7 0.87 0.5 7 5.1% 0.54 [-0.54, 1.61]
Ramirez-Campillo et al 2019 (G2) 1.32 0.2 7 0.98 0.2 7 4.5% 1.59 [0.33, 2.85]
Ramirez-Campillo et al 2019 (G3) 1.8 0.7 6 1.28 0.7 6 4.7% 0.69 [-0.49, 1.87]
Ramirez-Campillo et al 2019 (G4) 1.65 0.4 6 1.22 0.5 6 4.6% 0.88 [-0.33, 2.09]
Subtotal (95% CI) 181 181 84.6% 0.78 [0.31, 1.24]
Heterogeneity: Tau² = 0.63; Chi² = 58.49, df = 14 (P < 0.00001); I² = 76%
Test for overall effect: Z = 3.26 (P = 0.001)
Total (95% CI) 206 206 100.0% 0.81 [0.40, 1.21]
Heterogeneity: Tau² = 0.55; Chi² = 61.25, df = 17 (P < 0.00001); I² = 72%
Test for overall effect: Z = 3.89 (P < 0.0001)
Test for subgroup differences: Chi² = 0.17, df = 1 (P = 0.68), I² = 0%
-2 -1 0 1 2
Favours [Pre-Test] Favours [Post-Test]

b)

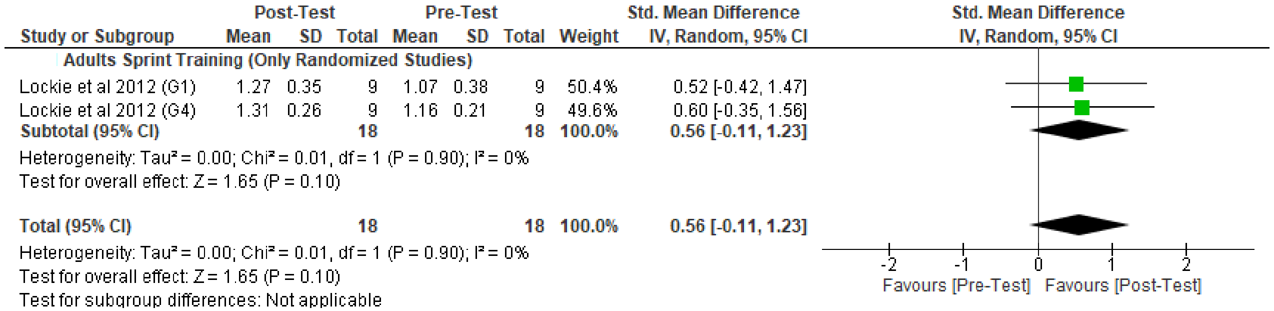
Post-Test
Pre-Test
Std. Mean Difference
Std. Mean Difference
Study or Subgroup Mean SD Total Mean SD Total Weight IV, Random, 95% CI IV, Random, 95% CI
Adults Sprint Training (Only Randomized Studies)
Lockie et al 2012 (G1) 1.27 0.35 9 1.07 0.38 9 50.4% 0.52 [-0.42, 1.47]
Lockie et al 2012 (G4) 1.31 0.26 9 1.16 0.21 9 49.6% 0.60 [-0.35, 1.56]
Subtotal (95% CI) 18 18 100.0% 0.56 [-0.11, 1.23]
Heterogeneity: Tau² = 0.00; Chi² = 0.01, df = 1 (P = 0.90); I² = 0%
Test for overall effect: Z = 1.65 (P = 0.10)
Total (95% CI) 18 18 100.0% 0.56 [-0.11, 1.23]
Heterogeneity: Tau² = 0.00; Chi² = 0.01, df = 1 (P = 0.90); I² = 0%
Test for overall effect: Z = 1.65 (P = 0.10)
Test for subgroup differences: Not applicable
-2 -1 0 1 2
Favours [Pre-Test] Favours [Post-Test]

c)

Study or Subgroup	Post-Test Mean	Post-Test SD	Post-Test Total	Pre-Test Mean	Pre-Test SD	Pre-Test Total	Weight	Std. Mean Difference IV, Random, 95% CI
**Adults Resistance Training (Only Randomized Studies)**								
Ciacci and Bartolomei 2018 (G1)	2.67	0.56	10	2.58	0.44	10	8.2%	0.17 [-0.71, 1.05]
Ciacci and Bartolomei 2018 (G4)	2.72	0.36	12	2.7	0.3	12	9.8%	0.06 [-0.74, 0.86]
Lockie et al 2012 (G2)	1.39	0.31	6	1.24	0.21	6	4.7%	0.52 [-0.64, 1.68]
**Subtotal (95% CI)**			**28**			**28**	**22.7%**	**0.19 [-0.33, 0.72]**
Heterogeneity: Tau² = 0.00; Chi² = 0.42, df = 2 (P = 0.81); I² = 0%								
Test for overall effect: Z = 0.72 (P = 0.47)								
**Youths Resistance Training (All Study Designs)**								
Chaabene et al 2019	1	0.2	18	1	0.2	18	14.8%	0.00 [-0.65, 0.65]
Ciacci and Bartolomei 2018 (G2)	2.6	0.47	10	2.4	0.37	10	7.9%	0.45 [-0.44, 1.34]
Ciacci and Bartolomei 2018 (G3)	2.86	0.36	9	2.62	0.43	9	7.0%	0.58 [-0.37, 1.52]
Ciacci and Bartolomei 2018 (G5)	2.69	0.46	8	2.52	0.32	8	6.4%	0.41 [-0.59, 1.40]
Ciacci and Bartolomei 2018 (G6)	2.21	0.25	9	2.01	0.39	9	7.0%	0.58 [-0.37, 1.53]
Murton et al 2021 (G1)	2.07	0.42	8	1.94	0.4	8	6.5%	0.30 [-0.69, 1.29]
Murton et al 2021 (G2)	2.22	0.47	8	1.97	0.35	8	6.2%	0.57 [-0.44, 1.58]
Orange et al 2019 (G1)	1.35	0.31	12	1.34	0.36	12	9.8%	0.03 [-0.77, 0.83]
Orange et al 2019 (G2)	1.28	0.2	15	1.16	0.18	15	11.7%	0.61 [-0.12, 1.35]
**Subtotal (95% CI)**			**97**			**97**	**77.3%**	**0.35 [0.07, 0.64]**
Heterogeneity: Tau² = 0.00; Chi² = 2.92, df = 8 (P = 0.94); I² = 0%								
Test for overall effect: Z = 2.42 (P = 0.02)								
**Total (95% CI)**			**125**			**125**	**100.0%**	**0.32 [0.07, 0.57]**
Heterogeneity: Tau² = 0.00; Chi² = 3.61, df = 11 (P = 0.98); I² = 0%								
Test for overall effect: Z = 2.47 (P = 0.01)								
Test for subgroup differences: Chi² = 0.27, df = 1 (P = 0.61), I² = 0%								

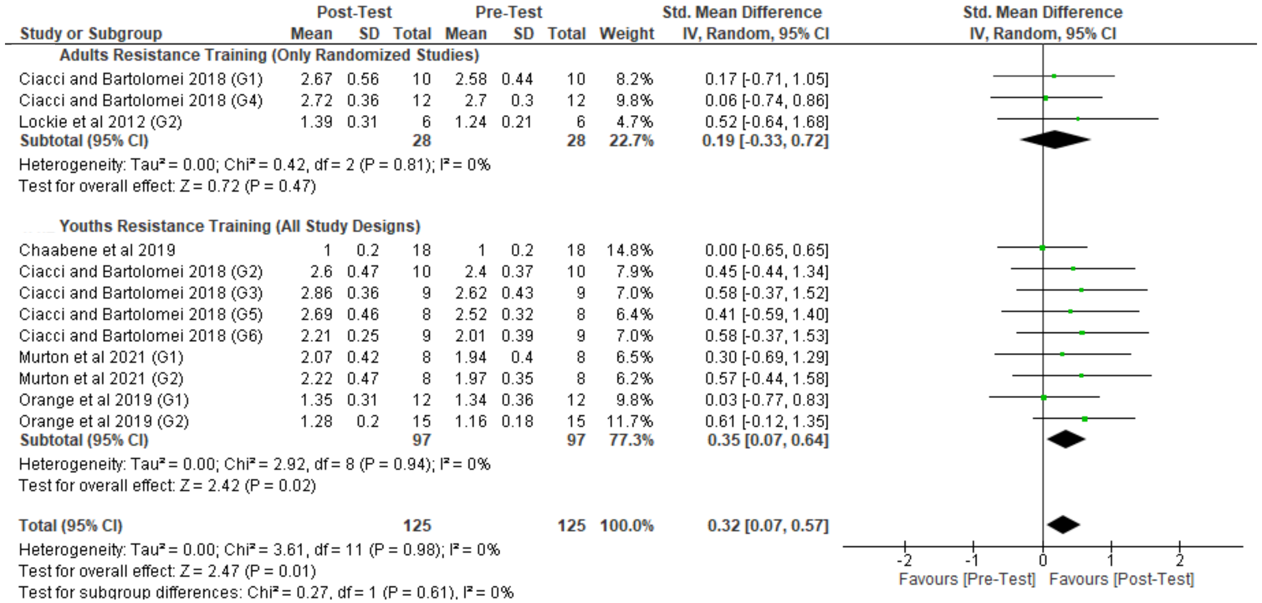

Figure S2. Forest plot of the overall standardized mean difference [95% CI] for each study (randomized and non-randomized) without control group (the size of the green dot corresponds to the weight of the study within the meta-analysis) with (**a**) a plyometric training intervention, (**b**) a sprint training intervention and (**c**) a resistance training intervention.
